# FLIM FRET Technology for Drug Discovery: Automated Multiwell-Plate High-Content Analysis, Multiplexed Readouts and Application in Situ**

**DOI:** 10.1002/cphc.201000874

**Published:** 2011-02-25

**Authors:** Sunil Kumar, Dominic Alibhai, Anca Margineanu, Romain Laine, Gordon Kennedy, James McGinty, Sean Warren, Douglas Kelly, Yuriy Alexandrov, Ian Munro, Clifford Talbot, Daniel W Stuckey, Christopher Kimberly, Bertrand Viellerobe, Francois Lacombe, Eric W-F Lam, Harriet Taylor, Margaret J Dallman, Gordon Stamp, Edward J Murray, Frank Stuhmeier, Alessandro Sardini, Matilda Katan, Daniel S Elson, Mark A A Neil, Chris Dunsby, Paul M W French

**Affiliations:** [a] Photonics Group, Department of Physics, Imperial College LondonLondon SW7 2AZ (UK), Fax: (+44) 2075947714; [b] Institute for Chemical Biology, Department of Chemistry, Imperial College LondonLondon SW7 2AZ (UK); [c] Clinical Sciences Centre, MRC, Imperial College LondonHammersmith Campus, London W12 0NN (UK); [d] Centre for Cell and Molecular Biology, Institute of Cancer ResearchLondon SW3 6JB (UK); [e] Mauna Kea Technologies9 rue d'Enghien, 75010 Paris (France); [f] Department of Surgery and Cancer, Imperial College LondonDu Cane Rd., London W12 0NN (UK); [g] Division of Cell and Molecular Biology, Department of Life Sciences, Imperial College LondonLondon SW7 2AZ (UK); [h] Department of Histopathology, Imperial College LondonDu Cane Rd., London W12 0NN (UK); [i] Pfizer Global Research and Development, Pfizer LimitedSandwich, Kent CT13 9NJ (UK); [j] Institute of Biomedical Engineering, Imperial College LondonLondon SW7 2AZ (UK)

**Keywords:** drug discovery, fluorescence lifetime imaging, FRET, high-throughput screening, proteins

## Abstract

A fluorescence lifetime imaging (FLIM) technology platform intended to read out changes in Förster resonance energy transfer (FRET) efficiency is presented for the study of protein interactions across the drug-discovery pipeline. FLIM provides a robust, inherently ratiometric imaging modality for drug discovery that could allow the same sensor constructs to be translated from automated cell-based assays through small transparent organisms such as zebrafish to mammals. To this end, an automated FLIM multiwell-plate reader is described for high content analysis of fixed and live cells, tomographic FLIM in zebrafish and FLIM FRET of live cells via confocal endomicroscopy. For cell-based assays, an exemplar application reading out protein aggregation using FLIM FRET is presented, and the potential for multiple simultaneous FLIM (FRET) readouts in microscopy is illustrated.

## 1. Introduction

Fluorescence is a ubiquitous readout of molecular localisation in the life sciences and has enabled the elucidation of many biological processes, particularly since the development of genetically expressed fluorophores has facilitated direct observation of signalling processes in live cells. Co-localisation of different fluorophores via fluorescence microscopy has provided evidence for protein interactions but this has been (diffraction) limited to the resolution of the microscope used. Although super-resolved imaging techniques[1] can break this diffraction limit, there is still a need for stronger direct evidence of protein interaction and this is afforded by Förster resonance energy transfer (FRET), which only occurs between fluorophores separated by less than ≈10 nm.[2] As well as being used to detect and monitor the binding of appropriately labelled proteins, either as an end point in fixed cells or as a dynamic process in live cells, FRET is also utilised in a range of genetically expressed intracellular biosensors of which the “Cameleon” calcium sensor[3] is the first and best known. A wide array of intramolecular FRET biosensors have now been reported, reading out, for example, calcium,[4] potassium,[5] chloride,[6] GTP,[7] IP3,[8] PIP2[9] and others.[10] In addition, there are “cleavage” sensors for which the FRET signal disappears upon activation, such as the calpain FRET sensor for monitoring calpain proteolytic activity,[11] to complement the vast range of intermolecular FRET readouts of protein interactions.

While there are many approaches[12] to detect FRET, the most widely used are probably spectral ratiometric imaging and fluorescence lifetime imaging (FLIM). Fluorescence lifetime measurements are particularly robust because they are usually independent of the fluorophore concentration, the excitation and detection efficiencies, and the impact of scattering and sample absorption. Spectral ratiometric measurements have the advantage that they are often easier to implement in terms of instrumentation and require fewer detected photons (and therefore shorter data acquisition times) than lifetime measurements. Their chief drawback is that they require calibration of the spectral response of the optical system, which can include the sample itself. This calibration is usually achieved through measurements of samples labelled with donor only and acceptor only as well as the sample under investigation, for example.[13] Once this is realised, spectral ratiometric measurements can then provide information concerning the effective FRET efficiency (i.e. the product of the actual FRET efficiency and the fraction of FRETing donor/acceptor molecules) and the relative total numbers of donor and acceptor molecules.[14, 15] With an independent measurement of the actual FRET efficiency of the interaction under investigation, spectral ratiometric FRET can then also provide the fractions of the FRETing donor and acceptor populations. The independent measurement of FRET efficiency (positive control) can be determined by a spectral ratiometric measurement of a FRETing sample exhibiting the same interaction with known stoichiometry (e.g. donor directly linked to acceptor) or by using another method, such as acceptor photobleaching or FLIM.[14, 15]

Fluorescence lifetime measurements are relatively robust in the presence of spectral crosstalk, being insensitive to donor–acceptor stoichiometry since it is only the donor fluorescence that is measured. They therefore do not require parallel spectral calibration measurements and are independent of the optical system, which makes them attractive for more challenging biological samples such as live animal models, for which the spectral properties can change in space and time. A determination of the mean fluorescence lifetime provides the effective FRET efficiency and fitting fluorescence lifetime measurements to a double-exponential decay model can provide an estimate of both the actual FRET efficiency and the fraction of FRETing donor population, which is suitable for quantitative assays such as dose–response curves. It should be noted, however, that fitting lifetime data to complex fluorescence decay models requires significantly more detected photons than measurements of single-exponential decay times, although this can be mitigated for FRET by using global analysis as discussed below. For this reason it is desirable to utilise fluorophores that exhibit monoexponential decay profiles.

FLIM thus provides a widely applicable means to read out FRET in order to map protein interactions and so to study cell signalling[16] and other processes. It can also be used to map other variations in the local fluorophore environment, for example utilising probes that can report on the concentration of analytes such as calcium,[17] or physical changes such as temperature[18] or lipid order.[19] The robust nature of FLIM readouts, including of FRET, means that they may potentially be translated along the drug-discovery pipeline from in vitro assays to animal models. In spite of this potential, however, FLIM has not yet made a significant impact on drug discovery. This is at least partly due to concerns about data acquisition times and to a lack of available instrumentation. Herein, we present a rapid automated optically sectioning FLIM multiwell-plate reader that we are developing for high content analysis (HCA) and illustrate its potential with an exemplar assay for aggregation of the Gag protein during the human immunodeficiency virus (HIV) cycle. To illustrate the future potential, we demonstrate the capability to simultaneously image multiplexed FLIM (FRET) readouts and to extend 3D FLIM and FRET to endoscopy and tomography of disease models.

The ability to read out protein interactions using FLIM and FRET in automated HCA could provide significant new opportunities for drug discovery and basic research, for example, providing the opportunity to utilise siRNA libraries for screening genetic perturbations with respect to signalling networks. A major obstacle to the application of FLIM to HCA has been the speed of data acquisition. To date, most FLIM experiments in cell biology have followed the first demonstration of FLIM microscopy,[20] and utilise time-correlated single photon counting (TCSPC)[21] implemented in a laser scanning confocal or multiphoton microscope. While this approach provides high-quality data, the imaging speed for this sequential pixel acquisition is limited by the constraints of single photon counting detection and by the nonlinear increase in photobleaching and photodamage that ensues as the power of the scanning laser beam is increased linearly. TCSPC has been implemented in a laser scanning multiwell-plate reader[22] but this instrument did not acquire fluorescence lifetime images, instead delivering a single lifetime measurement per well. Recently, an imaging multiwell-plate reader utilising multiphoton TCSPC FLIM was reported,[23] but this was for secondary measurements following identification of “hits” by steady-state polarisation-resolved anisotropy imaging since the FLIM was considered to be too slow for rapid measurements.

Wide-field FLIM achieves faster imaging rates than laser scanning FLIM with lower photobleaching due to the parallel pixel interrogation. The first demonstration of wide-field FLIM to read microarrays was demonstrated using time-gated imaging to improve the sensitivity for DNA profiling.[24] More recently, an automated instrument for unsupervised FLIM of multiwell-plate sample arrays was reported[25] that exploited the frequency domain (FD) fluorescence lifetime determination of FRET in a wide-field (non-sectioned) microscope. This provided an elegant demonstration of the potential of automated FLIM FRET and the opportunities afforded by statistical analysis of such FLIM array data. More recently, a wide-field FD FLIM plate reader has been applied to image post-translational modifications (tyrosine phosphorylation) in situ—specifically uncovering components that transduce signals from epidermal growth factor receptor.[26] While these wide-field FD FLIM instruments have been successful, they can be limited in signal-to-noise ratio by the lack of optical sectioning, which improves quantitative readouts by rejecting contributions from out-of-focus fluorescence (as is inherent in laser scanning confocal/multiphoton systems). FD FLIM systems can also be limited in speed by the need for sufficient temporal sampling of the fluorescence signal to avoid aliasing artefacts that can arise from pulsed excitation or nonlinearities in the excitation/detection modulation. This aliasing issue can, however, be addressed using special demodulation functions, as in the technique of *φ*^2^ FLIM.[15]

We recently demonstrated that wide-field time-gated imaging using a gated optical intensifier (GOI) can be combined with a Nipkow disc confocal microscope to provide high-speed optically sectioned FLIM that is significantly faster, for a given signal-to-noise ratio (*S*/*N*), than current TCSPC instrumentation. With this instrument we demonstrated the acquisition of time-gated FLIM FRET images of live cells labelled with FRET constructs at up to 10 frames per second.[27] Although wide-field time-gated FLIM generally yields a lower *S*/*N* ratio per photon emitted by the sample than TCSPC, the parallelism provides a greater *S*/*N* per unit acquisition time, which is important for studying dynamics and for higher-throughput applications. Wide-field time-gated imaging can facilitate FLIM with as few as three time-gates for monoexponential and five for double-exponential decay models in the presence of a background contribution. We previously published the first report of an automated high-speed optically sectioned FLIM multiwell-plate reader for HCA,[28] by utilising time-gated imaging via a home-built Nipkow disc microscope for automated (unsupervised) FLIM FRET with acquisition times of less than 10 s per well including sample motion, autofocus, cell finding and system calibration (or less than 16 min/96-well plate), and demonstrated its application to fixed and live cell imaging. Herein, we demonstrate that this approach can be implemented on a commercial wide-field multiwell-plate reader (GE Healthcare IN Cell Analyzer 1000) typically acquiring optically sectioned FLIM images in a few seconds and automatically reading a 96-well plate in ≈10 min, within which time we typically acquire several hundred photons per pixel.

Our in-house software controls the automated image acquisition and data analysis, including image segmentation and global analysis. Approximately 300 photons are sufficient to fit a fluorescence decay profile to a monoexponential decay model but not to more complex models. For many applications it is still useful to fit complex fluorescence decays to a monoexponential model and use the change in effective lifetime as an indicator, for example, of FRET. Alternatively, one can obtain more detailed information by applying global analysis to the same data. The simplest approach is global binning, which involves binning all the detected photons from a region of interest (ROI) and fitting the composite signal to, for example, a double-exponential decay model, to determine the fluorescence lifetime components under the assumption that the lifetimes are invariant across the ROI. This can be combined with automatic image segmentation to define the regions of interest. A more sophisticated approach is to apply global fitting,[29] which again assumes that the component lifetimes are invariant across the image but here the entire image data set is fitted in parallel to the model by minimising a global *χ*^2^. Global fitting is computationally intensive and so is normally applied in post-processing, while more immediate readouts are typically provided by fitting to a monoexponential decay model or by using analytical approaches such as rapid lifetime determination[30] or phasor analysis.[31, 32] Phasor analysis can also be applied to FLIM FRET data and provides useful graphical representations with no need for iterative fitting. It can be extended to include a “cluster-style” analysis of the phasor plot distribution that allows the signal at each pixel to be decomposed into FRETing and non-FRETing components, thus allowing the stoichiometry at each pixel to be determined.[32] Phasor analysis and global fitting techniques make the same basic assumption that the signal from each pixel can be described as a linear sum of two spatially invariant decay profiles. Phasor analysis imposes lower computational and memory requirements than global analysis, but a quantitative comparison of the information content, signal-to-noise performance and degree of bias for these two different approaches under practical experimental conditions has, to the best of our knowledge, not yet been undertaken.

The optically sectioning multiwell-plate reader described is being developed for FLIM and FRET assays and here we discuss its performance, including with respect to an exemplar application concerning the aggregation of HIV-1 Gag proteins in the late life cycle of the HIV-1 virion.[33, 34] HIV-1 Gag is the major structural protein within HIV-1 virions and is thought to interact with other viral proteins, the viral genome and with a large number of host cell factors, to orchestrate the formation of new virions at the plasma membrane of the target cell.[35] The expression of HIV-1 Gag alone within living cells leads to the formation of virus-like particles (VLPs), which provides a convenient means to study this late stage of the HIV cycle. By tagging HIV-1 Gag with either cyan fluorescent protein (CFP) or yellow fluorescent protein (YFP) at its C terminus, we are able to read out oligomerisation using FLIM FRET since co-transfection of Gag–CFP and Gag–YFP results in the production of VLPs that undergo FRET from Gag–CFP to Gag–YFP due to the close packing of Gag proteins within newly formed VLPs. While FRET has previously been used to monitor clustering of Gag proteins in this context,[36–38] we believe that this is a useful test of the utility of our new Nipkow FLIM multiwell-plate reader, noting that previous work was confined to confocal microscopy. The application of an automated FLIM plate reader could permit the screening of compounds designed to interfere with this important stage in the HIV life cycle.

For drug discovery, an automated FLIM multiwell-plate reader can be used to apply FRET to read out protein–protein interactions that can report on the impact of drug candidates on a cell-signalling network. While this is already a significant advance on the state of the art, such readouts would be more specific and informative if multiple components of cell-signalling networks could be probed simultaneously. Spectral ratiometric imaging has previously been used to simultaneously map two separate FRET readouts,[39, 40] but the spectral crosstalk remains a challenge when using this approach. To address this issue, we previously[41] multiplexed a FLIM FRET readout of Ras-Raf protein binding, using TagRed/mPlum as the donor–acceptor pair, with a spectral ratiometric readout of a “Cameleon” FRET probe[3] utilising CFP/YFP, to demonstrate simultaneous monitoring of two components of the epidermal growth factor (EGF) signal pathway in live COS-7 cells. We believe that this combination of fluorophores is widely applicable and offers the lowest degree of crosstalk for multiplexed FRET yet reported. Unlike ratiometric approaches to FRET, FLIM is able to make use of relatively dim acceptors such as mPlum—or even dark acceptors[42]—since only measurement of the donor is required. Indeed, the use of dark acceptors is desirable for FLIM FRET and can enable a higher density of multiplexed readouts. This earlier work was limited to wide-field microscopy by the equipment then available to us. We have now demonstrated an optically sectioned multiplexed FLIM microscope based on the approach of our earlier work[41] but using a Nipkow disc unit incorporating an electronically controlled dichroic changer and electronically controlled excitation and emission filter wheels. This enables interleaved FLIM acquisitions in two spectral channels, thus permitting the spatiotemporal mapping of two fluorescence lifetime readouts. For a first demonstration of this capability, we have simultaneously imaged the calcium transients induced in HEK293T cells following the addition of ionomycin using both a genetically expressed calcium sensor, Troponin TN-L15[43] labelled with CFP/Citrine, and a calcium-sensitive dye, GFP-Certified FluoForte (Enzo Life Sciences Ltd, UK). In the future we hope to implement multiplexed FLIM readouts on our multiwell-plate reader.

There is increasing concern that biological processes observed in monolayers of cultured cells are not necessarily reproduced in live model organisms, or human patients. This is of crucial importance to drug discovery and testing, but practical constraints—particularly the desire for convenient readouts—have nevertheless led most assays to be undertaken in conditions that are far removed from their native or intended physiological contexts. This has been particularly true of optical readouts, which have been widely used in high-throughput screening, but now there is an increasing trend to translate assays from cell monolayers to “more realistic” 3D cell and tissue cultures, to transparent live organisms that are amenable to genetic manipulation such as drosophila and zebrafish, and to small mammals. Imaging plays an important role in assays for such disease models although, to date, most of the readouts have been concerned with morphology and ultimate phenotypes, which can be difficult to directly correlate with cellular responses. We aim to translate FLIM and FRET along the drug-discovery pipeline and image functional changes in signalling networks that can be directly correlated with cell-based assays using the same readouts.

To this end we are developing tomographic FLIM instrumentation based on the technique of optical projection tomography (OPT),[44] which is analogous to X-ray computed tomography but uses visible radiation rather than X-rays and so requires the samples to be more or less transparent. This is usually achieved using a chemical clearing process that eliminates the scattering of visible radiation by refractive index matching throughout the sample using BABB (a 1:2 mixture of benzyl alcohol and benzyl benzoate) or other index-matching solutions. Our “tomoFLIM” approach utilises wide-field time-gated fluorescence imaging combined with a rotating sample stage. Reconstruction of 3D transmission and fluorescence images of transparent samples is realised using standard back-projection techniques to produce a series of time-gated images generated as a function of time delay after excitation. This can be processed to give 3D fluorescence intensity and lifetime distributions, which, in turn, can be used to map FRET in 3D throughout a sample. To date, we have demonstrated the ability to map FLIM in 3D in chemically cleared mouse embryos[45] and have demonstrated 3D FLIM of FRET in transparent phantoms. The prospect of reading out FRET-based assays using tomoFLIM in disease models such as zebrafish offers many opportunities in drug discovery, including lead validation and toxicology studies, as well as for molecular cell biology in general, but there remain some significant hurdles. The need for chemical clearing precludes the use of live samples and, unfortunately, the chemical clearing process itself appears to degrade the fluorescence of genetically expressed fluorescent proteins.[46] Very recently, however, we have succeeded in applying tomoFLIM to a fixed but not cleared zebrafish embryo labelled with enhanced green fluorescent protein (EGFP). This is an important step towards our ultimate goal of imaging live zebrafish.

Having identified drug candidates using cell-based assays, it is necessary to evaluate their performance, for example with respect to efficacy and toxicology, in animal models such as mice. Unfortunately such animals are far from transparent but the prospect of translating cell-based assays to animal models is stimulating increasing interest in addressing this challenge. Our tomoFLIM set-up can also be applied to imaging scattering samples but then the image reconstruction cannot be realised using back projection. Instead, it is necessary to take into account the multiple scattering of the detected photons and to use statistical diffuse light propagation algorithms to reconstruct the 3D fluorescence quantum efficiency and lifetime distributions based on the probable trajectories of the detected photons. Unlike OPT, which can deliver diffraction-limited images, diffuse fluorescence tomography (DFT) provides significantly decreased spatial resolution. Nevertheless, we have shown that DFT is able to reconstruct fluorescence lifetime distributions in scattering phantoms.[47] If the technique can be extended to live samples, such as genetically manipulated mice, then it would provide a further opportunity to correlate FLIM-based readouts, for example of cell-signalling networks, across the drug-discovery pipeline. We note that tomographic FLIM in diffuse media has received considerable attention recently, particularly with respect to the development of appropriate reconstruction algorithms (see, e.g., ref. [48]), but tomographic FLIM of FRET in a live subject is still yet to be reported.

For many investigations, it would be valuable to also obtain morphological information and to be able to image cell biological processes in vivo in live disease models, such as mice, with subcellular resolution. This can be realised using intravital microscopy, as is, for example, undertaken in neurological studies in live rodents,[49] or by using endoscopy. Although we believe that FLIM endoscopy provides a promising route to study signalling processes and disease mechanisms in vivo, as well as providing a new molecular imaging modality for clinical diagnosis, we note that there have been few reports of FLIM endoscopes since the first demonstration of FD FLIM applied to ex vivo and in vivo tissue imaging.[50] In the time domain, we have demonstrated wide-field time-gated FLIM in rigid[51] and flexible[52] endoscopes and note that, in general, the larger achievable fields of view and imaging speeds may make wide-field FLIM endoscopes more suitable for clinical diagnostic (screening) applications than laser scanning instruments, although the latter can provide optical sectioning and subcellular resolution to study signalling processes. For the latter application, we developed the first confocal laser scanning FLIM endomicroscope, which provides optically sectioned subcellular resolution that we applied to tissue autofluorescence and FRET in fixed cells.[53] We are not aware of any reports of multiphoton FLIM endoscopy but we note that multiphoton FLIM has been extensively used for intravital imaging of skin (see, e.g., ref. [54]) and has been applied to FRET studies of Alzheimer’s disease.[55]

Our confocal FLIM endoscope is based on a commercially available endomicroscope[56] (Cellvizio GI, Mauna Kea Technologies) to which we have added an ultrafast excitation laser and TCSPC detection to facilitate FLIM, thus realising acquisition times of a few seconds. This approach enables endoscopic confocal FLIM via a range of optical-fibre-bundle probes that either work in a “contact” imaging mode (i.e. working depth 0 μm) or use distal micro-optics to achieve optically sectioned imaging at a fixed working depth (e.g. from 20 to 80 μm). Subsequently a similar instrument, albeit limited to “contact imaging”, was demonstrated for FLIM of FRET in live cells immobilised in a gel-based matrix, and reporting FLIM acquisition times of 300 s.[57] Our earlier system[53] was assembled on a large optical table in a physics laboratory and we now describe a refined prototype intended for clinical imaging and animal experiments that is developed for integration into a trolley of area 70×100 cm^2^. To illustrate its potential for in vivo FLIM FRET studies, we applied it to live cells expressing EGFP and mCherry, either separately or joined by a short peptide linker to provide a FRET sample. Unambiguous FLIM FRET readouts in these live cells were obtained with acquisition times of a few seconds. To provide a real-time FLIM preview mode to guide clinicians and experimenters, we are now working to implement the first in, first out (FIFO) mode of TCSPC acquisition, which will also permit acquisition of larger image data sets. We note that the “time-tagged” photon detection associated with FIFO TCSPC will potentially enable us to use image registration techniques if FLIM of samples moving within the total acquisition time is required. We are also working on FLIM montaging techniques to combine multiple fields of view in a single data set.

## 2. Results and Discussion

### 2.1. HCA in an Optically Sectioned FLIM Multiwell-Plate Reader

**Figure 1:**
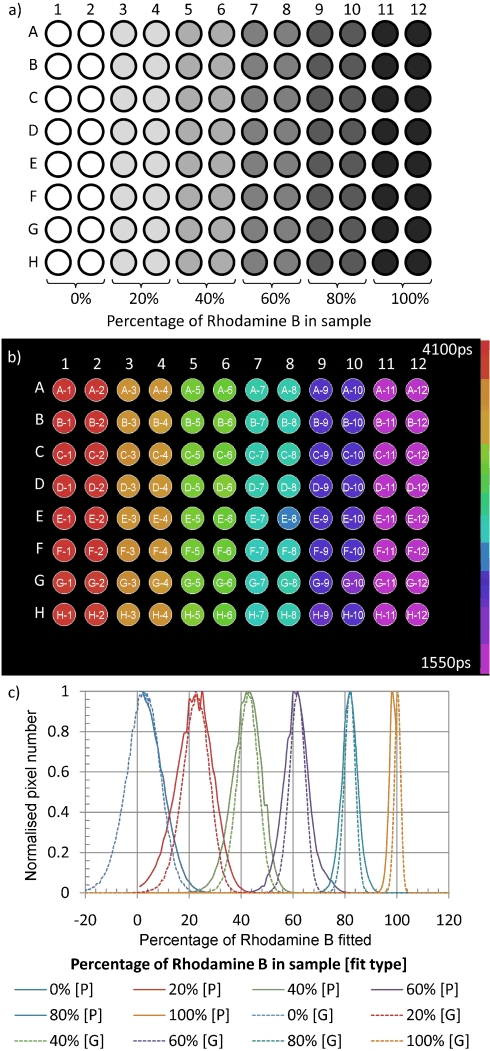
a) Plate map of mixtures of Rhodamine B and Rhodamine 6G (Rhodamine B percentage shown). b) False-colour fluorescence lifetime map of the mean of the lifetime histograms produced from per-pixel fitting with a single-exponential decay model. c) Histograms of percentage of Rhodamine B determined by per-pixel fitting to a double-exponential model (solid curves) and a global double-exponential fit to a subset of the data (dashed curves); [P]=predefined, [G]=global.

FLIM multiwell-plate readers may potentially be exploited for FRET and drug discovery and it is important to characterise their performance. For this purpose, we undertook a range of experiments applying our modified IN Cell Analyzer 1000 instrument to various dye and cell samples. Figure 1 a shows the map of a multiwell plate containing mixtures of the dyes Rhodamine B and Rhodamine 6G in various ratios, which we imaged by acquiring FLIM images from four fields of view per well. As a way of initially visualising the data, we fitted a single-exponential decay model to all pixels acquired from each well and then calculated the mean lifetime for that well (see Figure 1 b). We then used two double-exponential decay analysis methods to illustrate the capability to determine the ratio of different fluorophore components, as illustrated in Figure 1 c. In the first method, we determined the lifetimes of Rhodamine B and Rhodamine 6G from the 100 and 0 % Rhodamine B wells, respectively, by taking the mean of a single-exponential fit to each image pixel. We then performed a double-exponential fit to each pixel using all of the pixels from a particular dye ratio (i.e. over 16×4 FLIM data sets), with the two lifetimes fixed to the values determined in the previous step. The resulting histograms of the percentage of Rhodamine B decay component are shown as the solid curves in Figure 1 c. In the second method, we took a subset of the data consisting of one FLIM image per dye ratio (i.e. six images) and used global double-exponential fitting to analyse all of the pixels in the data sets simultaneously. The use of a subset of the data for the global fitting was due to computer memory constraints for the global fitting algorithm (see the dashed curves in Figure 1 c).

**Figure 2:**
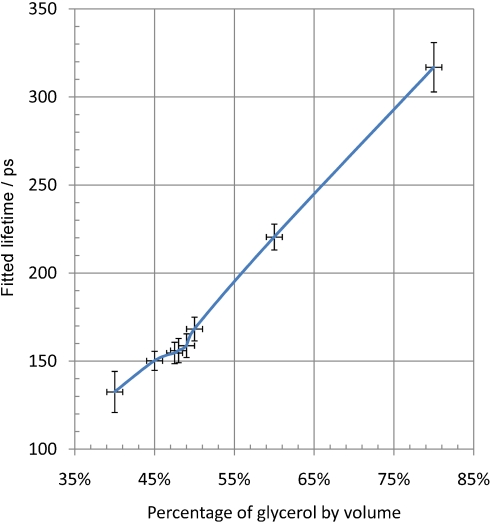
Mean fluorescence lifetime ± standard deviation of histogram ([glycerol]±1 % error).

The ability to resolve small fluorescence lifetime differences was also investigated using a dye, *trans*-4-[4-(dimethylamino)styrl]-1-methylpyridinium iodide (DASPI), the fluorescence lifetime of which is known to vary with the solvent viscosity.[58, 59] Figure 2 shows the variation of the mean fluorescence lifetime per well (calculated by fitting pixel-wise to a monoexponential decay model and then taking the mean of the resulting histogram) obtained for DASPI dissolved in a series of composite solvents comprising mixtures of ethanol and glycerol ranging from 40 % to 80 % glycerol. The temporal instrument response function (IRF) used for this fit was determined using the attenuated excitation laser to directly illuminate the gated intensifier. Figure 2 illustrates that, while our FLIM multiwell-plate reader was set to acquire data with a gate width of 2000 ps, it can be used to measure the fluorescence decay of fluorophores with much shorter lifetimes, due to the steep edges of the gated optical intensifier (GOI) time-gate, and can distinguish lifetime differences less than ≈20 ps. We note that the fluorescence lifetime of DASPI dissolved in water is less than 150 ps, as determined from cuvette-based measurements using our home-built fluorometer,[60] and DASPI thus provides a convenient means to obtain an IRF for a range of FLIM instrumentation.

**Figure 3:**
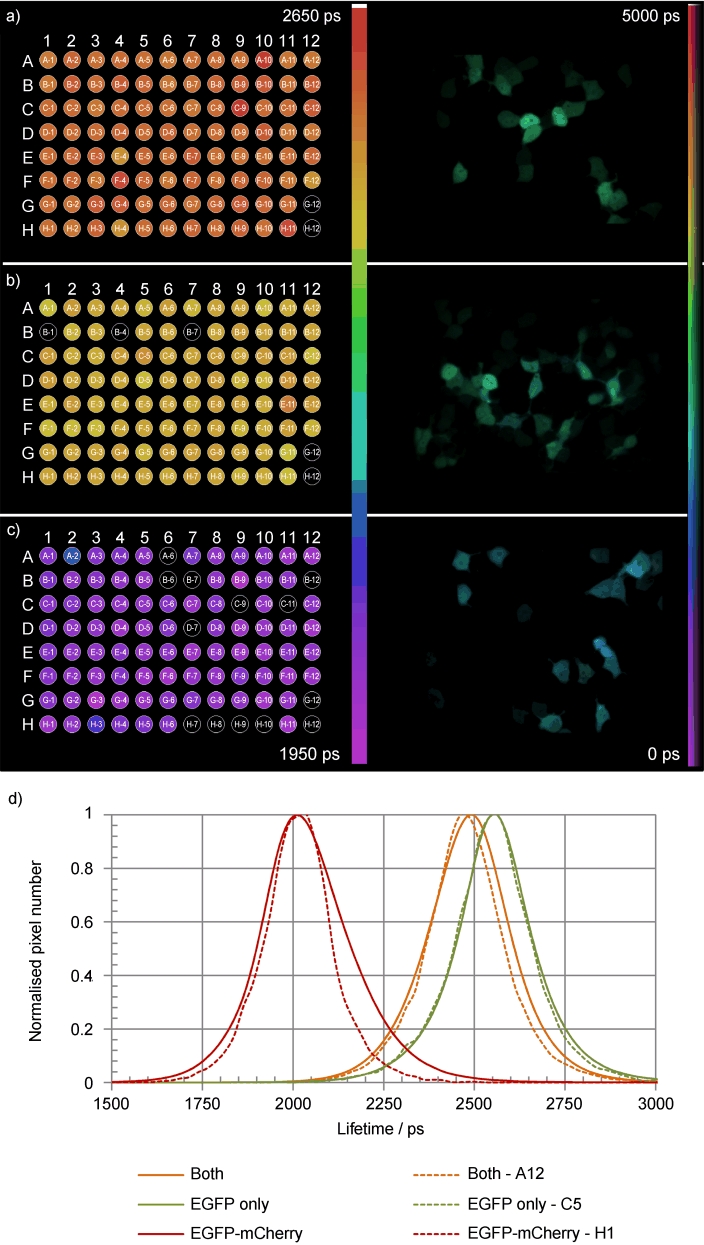
Mean well-plate lifetime maps and representative fluorescence lifetime images of live cells expressing a) EGFP (280 fields of view acquired), b) EGFP–mCherry and EGFP (272 fields of view acquired) and c) EGFP–mCherry (245 fields of view acquired). Black wells in (a–c) indicate that the acquisition algorithm was unable to locate sufficient cells in these wells. d) Lifetime histograms over each whole plate (solid curves) and exemplar individual well (dashed curves) data sets.

To characterise the performance of this FLIM multiwell-plate reader with live cells, we produced three multiwell plates containing HEK293T cells expressing EGFP only, an EGFP–mCherry tandem FRET construct and a mixture of EGFP and the EGFP–mCherry construct. Figure 3 shows maps of the mean fluorescence lifetime across each well (with lifetimes averaged over up to three fields of view within a well) and exemplar fluorescence lifetime images (for which the false-colour lifetime map has been merged with the fluorescence intensity map) for 96-well plates presenting cells transfected with EGFP only (Figure 3 a), both EGFP and the EGFP–mCherry construct (in an unknown stoichiometry; Figure 3 b) and only the EGFP–mCherry construct (Figure 3 c). It should be noted that the fluorescence lifetime colour scale used for the mean lifetime maps has been optimised for maximum contrast and is not the same as that used for the fluorescence lifetime images. In each case the data from each pixel were fitted to a monoexponential decay model using an IRF obtained by imaging DASPI in ethanol. For each labelling configuration, Figure 3 d shows the corresponding fluorescence lifetime histograms for all the wells from each well plate and for a single representative well from each of the well plates. The small difference between the solid and dotted histograms indicates the relatively low inter-well variation in our measurements. The larger difference between the single-well and multiwell histograms for the EGFP–mCherry construct may be due to the presence of incomplete and/or immature mCherry protein in some cells in the multiwell plate. The blank wells are those where the multiwell-plate reader failed to find sufficient cells to image and hence no data were acquired in these wells.

The time for each multiwell-plate acquisition varies depending on the number of fields of view successfully acquired, the number of time gates used (seven in this case, with one being an offset image and one providing a check against sample motion) and the integration time for each time-gated image (0.2 s in the case of the EGFP-only plate, 3 s for the linked EGFP–mCherry plate and 0.2 s for the plate with both constructs expressed). The average FLIM acquisition times per field of view for the EGFP-only, linked and mixed FP construct plates (including sample motion and autofocusing) were 4.2, 23.6 and 4.1 s respectively, which give equivalent 96 field-of-view acquisition times of 6.7, 37.8 and 6.7 min, respectively.

This FLIM FRET data set was also analysed using global fitting. First, six fields of view of cells expressing EGFP only were globally fitted using a single-exponential decay model, which resulted in a lifetime of 2516 ps for the EGFP (*τ*_1_). We then globally fitted data from six fields of view (three from EGFP–mCherry and three from EGFP and EGFP–mCherry) using a double-exponential decay model of the form [Eq. (1)]:


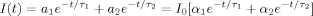
(1)

where *a*_1_ and *a*_2_ are the pre-exponential factors, *α*_1_ and *α*_2_ are the normalised pre-exponential factors (i.e. *α*_1_+*α*_2_=1), *I*_0_ is the initial fluorescence intensity and *τ*_1_ and *τ*_2_ are the fluorescence decay times. Global fitting returned the global lifetimes of the non-FRETing donor (*τ*_1_), FRETing donor (*τ*_2_) and the fractions of non-FRETing donor and FRETing donor contributions (*α*_1_, *α*_2_) in each pixel while fixing the value of the non-FRETing EGFP donor, *τ*_1_, to 2516 ps. This global analysis was limited to data from a total of six fields of view by the available computer memory and the results are shown in Table 1.

**Table 1:** Results of global analysis of cells expressing EGFP, EGFP–mCherry and both EGFP and EGFP–mCherry. Six EGFP fields of view were globally fitted using a single-exponential decay (top row). The resulting lifetime (*τ*_1_) was then fixed for the subsequent global double-exponential decay analysis (bottom two rows)

	Mean *α*_1_	Global *τ*_1_	Mean *α*_2_	Global *τ*_2_
EGFP only	1.00	2516	N/A	N/A
EGFP–mCherry construct	0.53		0.47	
EGFP and EGFP–mCherry constructs	0.90	2516 (fixed)	0.10	1068 (fitted)

N/A: not applicable.

We also performed a global double-exponential fit where both fluorescence lifetimes were free and in this case obtained a slightly longer value for the non-FRETing donor of *τ*_1_=2572 ps. For comparison, we obtained a mean value of *τ*=2559 ps for the per-pixel single-exponential fit of EGFP described above in Figure 3.

**Figure 4:**
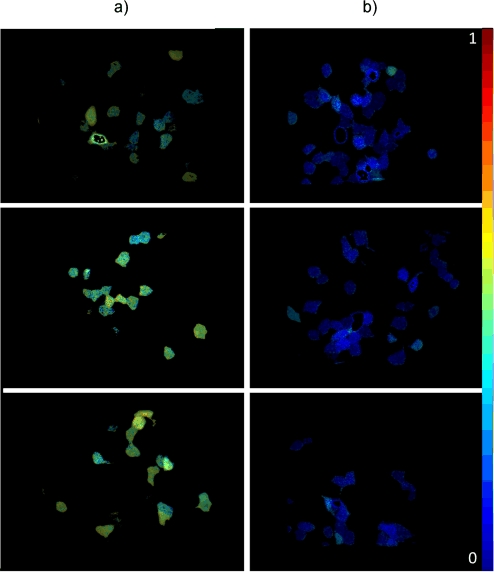
Fraction of FRETing donor population, *α*_2_, calculated using a global double-exponential fit with EGFP component fixed (*τ*_1_=2516 ps) for cells expressing a) EGFP–mCherry only and b) EGFP and EGFP–mCherry.

Figure 4 shows the FRETing population fraction, *α*_2_, calculated for three representative wells for each of the two labelling configurations that were fitted using the global double-exponential decay analysis. All six images were fitted globally with the EGFP lifetime fixed (*τ*_1_=2516 ps). It can be seen in Figure 4 a that the cells expressing the EGFP–mCherry tandem construct exhibit a significant proportion (47 %) of the *τ*_2_ component and the mixture represented in Figure 4 b shows the *τ*_1_ component (i.e. EGFP alone) dominating at ≈90 %, which is in agreement with the lifetime histograms of Figure 3 d. The presence of a significant *τ*_1_ component in Figure 4 a may be due to partial expression and/or maturation of the mCherry in the tandem construct.

### 2.2. Automated Multiwell-Plate FLIM Applied to Study HIV-1 Gag Oligomerisation

As an exemplar biological assay, this automated Nipkow FLIM multiwell-plate reader was applied to FLIM FRET of HIV-1 Gag protein oligomerisation in HIV-1 VLPs within live HEK293T cells. The HIV-1 Gag protein has been extensively studied,[33–38] and here we use it to demonstrate the potential of our multiwell-plate reader. As previously discussed, FRET provides a readout of protein aggregation even if proteins labelled with donor and acceptor fluorophores are expressed with a random stoichiometry, since some donors and acceptors should then find themselves in close proximity. The increase in FRET can be measured by observing the decrease in CFP donor lifetime when interacting with YFP acceptors in the context of the resulting VLP structure.

**Figure 5:**
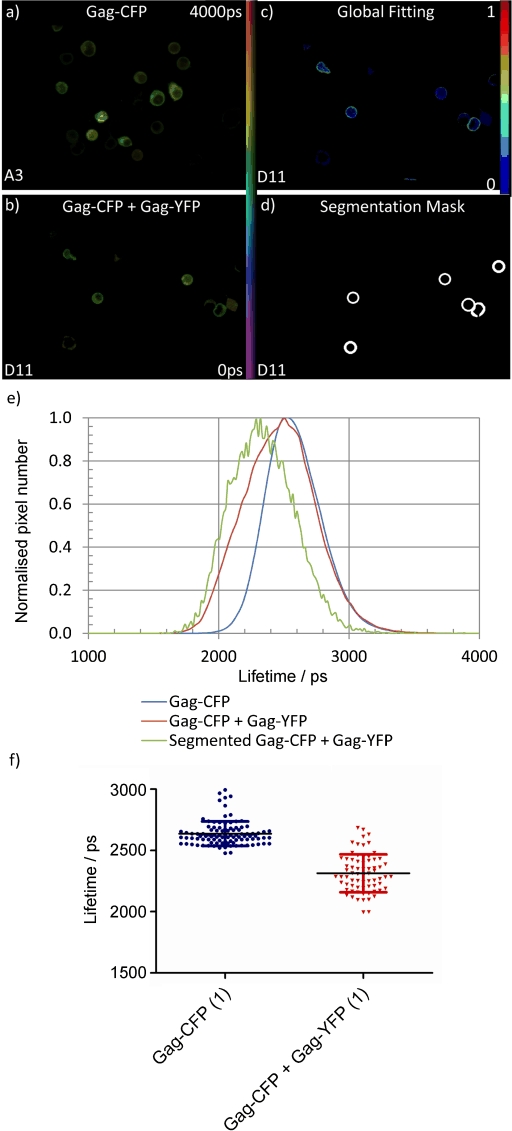
a,b) Fluorescence lifetime images of HEK cells with HIV virions expressing a) Gag–CFP only and b) Gag–CFP and Gag–YFP in an unknown ratio. c) Map showing the fraction of the short-lifetime component (*τ*_2_=1586 ps) obtained from globally fitting a double-exponential decay across the whole field of view with the first component fixed to 2580 ps. d) Image segmentation mask manually generated from the fluorescence intensity image in (b). e) Mean lifetime histograms corresponding to all pixels from 48 Gag–CFP-only wells (blue line), to all pixels from 47 Gag–CFP/Gag–YFP wells (red line) and to only pixels included in the manual segmentation mask over all 47 Gag–CFP/Gag–YFP wells (green line). f) The per-ROI lifetime analysis of all cells in all wells, that is, each membrane region is segmented and binned to produce a single decay profile, which is then fitted with a single-exponential decay model to calculate the mean lifetime.

In our first experiments we imaged a multiwell plate containing HEK293T cells in which half the wells were transfected with HIV-1 Gag–CFP (donor only), while the other half was transfected with HIV-1 Gag–CFP and HIV-1 Gag–YFP (donor and acceptor). The Gag–CFP fusion protein has been characterised in detail by numerous laboratories and it has been shown that the presence of CFP—which, in isolation, is noted to be a weak dimer[61]—does not affect its normal aggregation.[36, 62–64] These cells were grown, transfected and plated as described in the Experimental Section. The FLIM multiwell-plate reader was set up to excite the Gag–CFP with a supercontinuum source and a 434/17 nm excitation filter, and the cells were imaged through a 482/35 nm emission filter. The total acquisition time was ≈7 s per well, with the whole plate being read out in approximately 11 min. Figure 5 a and b show representative lifetime images of wells containing these cells, for which the FLIM data were fitted on a per-pixel basis to a monoexponential decay model because there were insufficient detected photons to fit pixel-wise to a more complex profile. A modest lifetime reduction (±standard deviation) from (2580±78) to (2470±113) ps is observed in the average CFP donor lifetime for the per-pixel single-exponential lifetime analysis. Figure 5 c shows the same field as Figure 5 b but using global analysis to fit a double-exponential decay to the same FLIM data. For this analysis, the lifetime of the non-FRETing donor was globally fixed to *τ*_1_=2580 ps, which was the value obtained from the single-exponential fit to the donor-only data. This global analysis shows that the FRETing CFP, indicated by the shorter fluorescence lifetime, occurs near the cell membrane where the Gag protein is expected to aggregate[34] and reported a global value of *τ*_2_=1586 ps.

To increase the sensitivity to changes in the CFP donor lifetime, the manually generated segmentation mask shown in Figure 5 d was applied to select only the pixels in or near the cell membrane and to exclude pixels from the cytoplasm. Previous work has shown that up to 75 % of myr(+)Gag (myr=myristoyl group) is found in the membrane.[36, 62, 65] This segmentation approach causes the apparent average CFP lifetime to decrease to (2340±90) ps and is illustrated by the green curve in Figure 5 e. To further increase the accuracy of the analysis, the spatially binned fluorescence decay from each cell membrane was then fitted to a single-exponential decay in a “per-ROI” analysis and Figure 5 f shows the resulting lifetime scatter plots. The per-ROI analysis was then performed for the entire multiwell-plate data set, which yielded an average membrane CFP lifetime of (2637±99) ps (*N*=138) for Gag–CFP and of (2313±155) ps (*N*=99) for cells co-transfected with Gag–CFP+Gag–YFP.

To investigate this readout of Gag oligomerisation further, we also undertook measurements of cells expressing a mutated Gag protein for which a glycine within the matrix region of Gag was deleted. This deletion prevents the addition of a myristoyl group to the N terminus of Gag, a normal post-translational modification that is thought to be necessary for Gag to bind to the plasma membrane.[66] Since it is at the plasma membrane that the Gag oligomerisation is thought to take place, the modification of Gag to make it “myristoyl group negative” (referred to here as myr(−)Gag) should therefore provide a biological control for Gag aggregation at the plasma membrane. The lack of aggregation at the membrane for myr(−)Gag was confirmed by confocal microscopy (data not shown).

**Figure 6:**
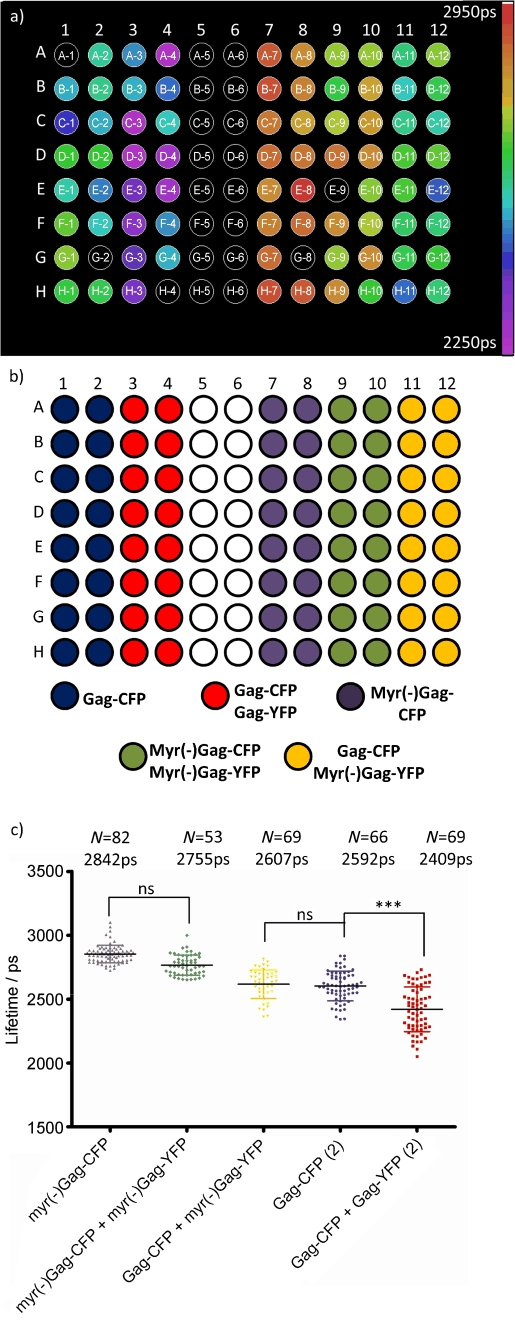
a) Map of measured mean per-ROI CFP lifetimes, b) multiwell-plate map and c) plot of per-ROI CFP lifetimes for assay of aggregation of a range of different HIV Gag proteins.

A multiwell map plate of cells expressing a variety of combinations of Gag–CFP, myr(−)Gag–CFP, Gag–YFP and myr(−)Gag–YFP is shown in Figure 6 a. This multiwell plate was imaged in an average of 9 s per well, which corresponded to reading the whole plate in just over 14 min. As described above, each cell was manually segmented to extract only pixels near to the plasma membrane where FRET is expected to occur. For cells expressing Gag–CFP and Gag–YFP, we observe a significant decrease in donor lifetime and this can be seen in both Figure 5 and Figure 6. The changes in lifetime observed in the other columns are consistent with the deletion of the first glycine in the matrix portion of Gag preventing myristoylation and therefore inhibiting membrane association and oligomerisation of this protein. More specifically, for cells expressing myr(−)Gag–CFP and myr(−)Gag–YFP we expect almost no Gag protein binding at the plasma membrane and so this acts as a biological control for negligible FRET (columns 1 and 2 of Figure 6 c). It is expected that a small fraction of membrane-bound Gag–CFP could dimerise with myr(−)Gag–YFP leading to FRET, although we believe this effect to be small since no significant difference is observed compared to Gag–CFP alone (columns 3 and 4 of Figure 6 c). The lifetime of Gag–CFP can also be modified by homoFRET or refractive index effects at the membrane, as discussed below.

We note that both Gag and myr(−)Gag and are believed to be able to dimerise in the cytosol and we therefore could expect the cytosolic Gag proteins to also present reduced CFP lifetimes due to FRET. In the case of myr(+)Gag transfected cells, the image segmentation to select only the region corresponding to the cell membrane will mean that the detected signal is predominantly from membrane-bound protein due to its higher concentration. In the case of myr(−)Gag transfected cells, the signal from a similar membrane ROI should be predominantly from cytosolic protein and one would therefore also expect to see a decrease in lifetime for the cells transfected with both myr(−)Gag–CFP and myr(−)Gag–YFP compared to just myr(−)Gag–CFP. A small decrease is observed between columns 1 and 2 of Figure 6 c, although it is not statistically significant. This suggests that the dimerisation of the cytosolic Gag protein is weak compared to the multimerisation at the membrane.

It is interesting to note that, although the cells expressing myr(−)Gag–CFP show a lifetime that is consistent with previously reported values for CFP, the cells expressing the normal Gag–CFP construct show a reduced lifetime, where the majority of their fluorescence is observed at the membrane. This lifetime reduction is most likely to be due to the formation of VLPs that concentrate the Gag–CFP molecules in close proximity at the membrane. It has previously been shown that the lifetime of CFP, with its multiple fluorescing components, can be decreased by homoFRET as well as by the effect of refractive index change at the membrane.[67] A further factor could be an interaction of HIV-1 Gag with host cell proteins, of which Tetherin and Tsg101 are potential candidates. The lifetime reduction of CFP by homoFRET is associated with the presence of the multiple CFP species that result in its complex decay profile, and should not be observed if a monoexponential decaying fluorophore could be employed. We are therefore working to replace CFP with teal fluorescent protein (TFP)[68] or mTurquoise,[69] which we believe will facilitate more quantitative readouts of FLIM and FRET.

### 2.3. Multiplexed Optical Sectioned FLIM Readouts

**Figure 7:**
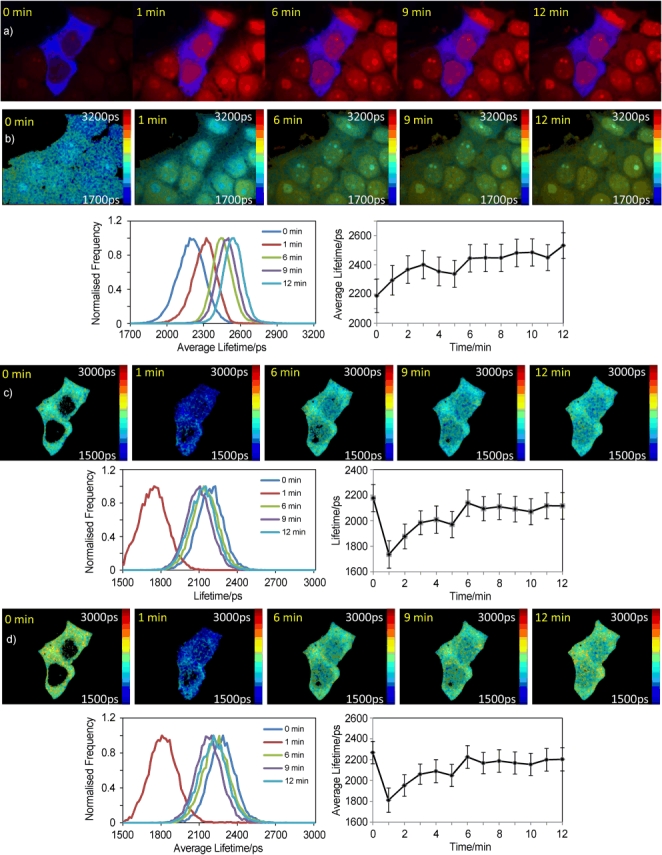
Multiplexed FLIM of calcium sensors. a) Overlay of intensity images of CFP in TN-L15 (blue) and GFP-Certified Fluoforte (red). b) FLIM images of GFP-Certified Fluoforte at selected time points of the measurement. Below the images are histograms of the average lifetimes (left) and the time variation of the mean lifetime (right). c) FLIM images of CFP in TN-L15 obtained using a single-exponential fit, which represent the average lifetimes at selected time points of the measurement. The graphs show the corresponding lifetime histograms (left) and the time variation of the mean lifetime (right). d) The same data as in (c) but instead fitted using a global analysis in which the ratio of the pre-exponential decay parameters is fitted as a global parameter across the whole FLIM time series (see text). The graphs show histograms of the average lifetimes (left) and the time variation of the mean lifetime (right). All images were analysed using 4×4 binning.

We demonstrated the capabilities of our optically sectioned multiplexed FLIM microscope by interleaved time-lapse imaging of live cells simultaneously labelled with two calcium probes following stimulation with 20 µm ionomycin and 12 mm calcium solutions. HEK293 cells were transfected with the genetically expressed biosensor TN-L15, for which changes in calcium levels are read out by FRET between the CFP and Citrine fluorophores, and also with a calcium-sensing dye, GFP-Certified FluoForte, which is conventionally read out using its fluorescence intensity. The emission profile of the latter is spectrally separate from those of CFP and Citrine, thus making it useful for multiplexing with the many CFP/YFP FRET biosensors that are available. As well as the change in fluorescence intensity with variations in calcium concentration, the fluorescence lifetime of GFP-Certified FluoForte also changes. We are still in the process of characterising the fluorescence lifetime readout of this probe, which we will publish in detail elsewhere, but it serves here to illustrate the ability to acquire FLIM data in two channels. Figure 7 shows the series of fluorescence lifetime images acquired in each channel following stimulation by ionomycin.

In a similar manner to the work of Wilms and Eilers[70] using different calcium dyes, we applied a double-exponential decay model to describe the fluorescence decay profile of GFP-Certified FluoForte corresponding to a mixture of unbound and calcium-bound dye molecules. Due to the limited fluorescence signal available, which was constrained by photobleaching considerations, we applied global fitting, for which the two lifetime components (*τ*_1_ and *τ*_2_) were linked across the whole FLIM time series. The fitting algorithm returned values of *τ*_1_=3139 and *τ*_2_=588 ps, which we attribute to the bound and unbound states of the dye, respectively; the results of this analysis are shown in Figure 7 b. The FLIM map pre-stimulation (0 min) shows a significantly shorter lifetime than the post-stimulation FLIM maps, as can also be seen in the associated histograms and plots of mean lifetime. The error bars on the plots of mean lifetime (right-hand plot in Figure 7 b) are the standard deviation of the lifetime distribution, and so indicate both the variation in lifetime due to photon statistics as well as spatial variation in lifetime across each image. Therefore these error bars represent an upper limit on the uncertainty of the measured mean lifetime.

For the TN-L15 data, the fluorescence decay of CFP is known to have a complex exponential decay profile and this greatly complicates the data analysis. As an initial approximation, we chose to fit a single-exponential decay to each pixel to estimate the mean fluorescence lifetime, as shown in Figure 7 c. A significant decrease in fluorescence lifetime of the TN-L15 FRET construct is observed between pre-stimulation (0 min) and at 1 min post stimulation. For comparison, we also performed a global analysis of the TN-L15 data. We made the assumption that the CFP chromophore exists in two different species—each with different lifetimes—and that the ratio of these two species is maintained throughout the experiment. We therefore performed a global analysis in which the ratio of the two species remained constant over the whole time series. This approach yielded the same trend as the single-exponential decay approximation although it provided slightly different values for the average lifetime.

We believe that all these readouts and analyses are consistent, and demonstrate that the stimulation of the cells by ionomycin and the consequent increase of intracellular calcium levels have resulted in changes in fluorescence lifetime of both the CFP donor in the TN-L15 FRET construct and the calcium-sensing dye that our system has been able to follow. This technique may be extended to two multiplexed FLIM readouts of spectrally distinct FRET biosensors, which utilise fluorophores such as the combination of CFP/YFP and TagRed/mPlum reported in our previous paper.[41] We would caution against the use of fluorophores such as CFP, however, which present complex exponential decay profiles and can lead to fluorescence lifetime artefacts, for example due to differential photobleaching of different species or to difficulties in fitting such complex decay data. It is clearly desirable to work with donor fluorophores exhibiting monoexponential decay profiles, although we note that these are relatively uncommon and that even EGFP exhibits a contribution (≈10 %) from a second component.[71] Unfortunately, most FRET biosensors reported to date utilise the CFP/YFP FRET pair and so we believe that it is highly desirable to clone new variants with monoexponential donors such as mTurquoise.[69] For multiplexed readouts, it is important to develop FRET probes that emit in spectral windows distinct from those of CFP/YFP. It would also be interesting to take advantage of “dark” acceptors such as REACH,[42] which can help minimise spectral crosstalk.

### 2.4. Tomographic FLIM Using Optical Projection Tomography

**Figure 8:**
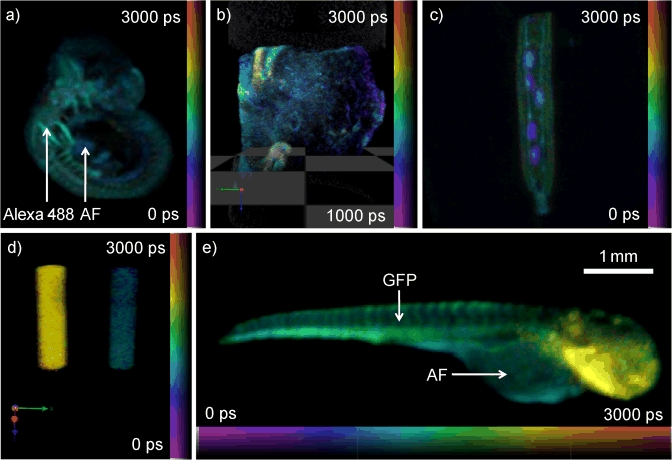
3D FLIM-OPT images of a) mouse embryo with Alexa-488-labelled neurofilaments; b) cleared but unstained ex vivo tissue section; c) cleared but unstained silique of Arabidopsis; d) wells containing cytosol preparations of TN-L15 with CaCl_2_ added to the right-hand well; and e) fixed but not cleared FLI:GFP zebrafish. AF=autofluorescence.

Tomographic FLIM of transparent samples has been implemented using the OPT set-up described in the Experimental Section. OPT was originally developed as a tool for 3D imaging of approximately millimetre- to centimetre-scale samples such as mouse embryos, and Figure 8 a shows a FLIM-OPT image of a mouse embryo that has been optically cleared using BABB, fixed in formaldehyde and the neurofilaments labelled with an Alexa-488-conjugated antibody.[45] Upon spectrally filtered supercontinuum excitation at (485±10) nm and tomographic image reconstruction, the Alexa-488-labelled neurofilaments presented a lifetime of (1360±180) ps and autofluorescence, observed in the unlabelled heart and dorsal aorta, presented a fluorescence lifetime of (1030±135) ps. The data acquisition entailed acquiring time-gated images at 360 projection angles, which required a total of 800 s. This timescale compares favourably to that required to obtain 3D image data by conventional serial sectioning and sequential imaging of extended samples, such as tissue biopsies, and FLIM-OPT appears to have potential as a tool for histopathology. Figure 8 b shows a 3D fluorescence lifetime image of a cleared but unstained sample of lung tissue, for which the lifetime contrast is provided by the tissue autofluorescence. This label-free optical sectioning approach may find applications where 3D histopathology is required.

Autofluorescence can also be exploited for label-free contrast in plant samples. Figure 8 c shows a 3D fluorescence lifetime image of a cleared but unstained silique from an Arabidopsis plant in which the fluorescence lifetime clearly contrasts the silique and seeds. As already discussed, while the optical clearing process is necessary for OPT and is generally compatible with fluorescence labelling, it is found to degrade the signals from fluorescent proteins. We are currently working to develop tomographic FLIM readouts of FRET and Figure 8 d illustrates a proof-of-principle FLIM FRET OPT experiment, showing a 3D fluorescence lifetime image of a (transparent) silicone phantom with two wells containing cytosolic preparations of HEK293T cells expressing the calcium sensor, TN-L15. CaCl_2_ was added to one of the wells, which resulted in an increased FRET signal, as indicated by the 550 ps difference in fluorescence lifetimes. This motivates us to apply FLIM to read out FRET sensors in small animals such as mouse or zebrafish embryos, but unfortunately the chemical clearing process often precludes the use of fluorescent proteins unless they can be re-labelled post-clearing, for example, by using an antibody to the fluorescent protein. Fortunately, however, we have recently observed that at 3 days post-fertilisation, zebrafish embryos are sufficiently transparent for the direct application of (FLIM) OPT. Figure 8 e shows a 3D fluorescence lifetime image of a fixed but uncleared transgenic (FLI:GFP) zebrafish in which the vasculature is genetically labelled with EGFP. Autofluorescence from the yolk sack is also apparent with lifetime contrast. We are currently working to extend this to FLIM-OPT of live zebrafish and believe that this will create many new opportunities to use FLIM FRET to study cell-signalling pathways for drug discovery and biomedical research.

### 2.5. Microconfocal FLIM Endoscopy

**Figure 9:**
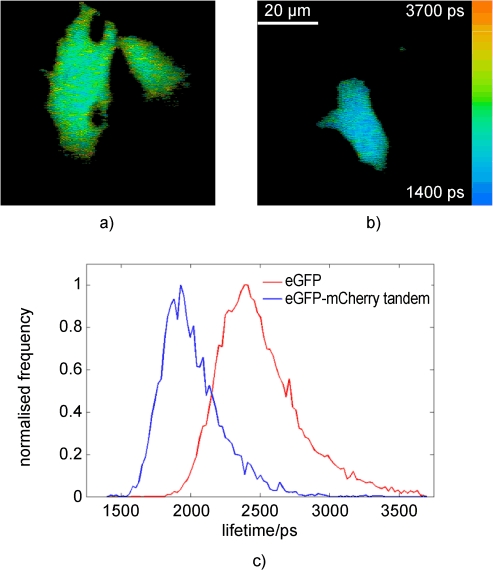
Confocal FLIM endomicroscopy images of COS-7 cells expressing a) EGFP and b) EGFP–mCherry tandem FRET construct; c) the corresponding fluorescence lifetime histograms.

To demonstrate the potential of confocal FLIM endomicroscopy for imaging spatiotemporal dynamics of cell-signalling processes, we applied it to a FRET model in live COS-7 cells that were transfected with EGFP or an EGFP–mCherry tandem FRET construct. Figure 9 shows the optically sectioned FLIM images acquired, with the corresponding lifetime histograms, using the confocal FLIM endomicroscope to image the cells in culture. The FLIM data were fitted to a single-exponential decay model and the clear decrease in the lifetime of the EGFP donor expected for FRET in the tandem construct is apparent. The data for Figure 9 a and b were acquired in 2 s and analysed with 5×5 pixel binning (SPCImage binning factor=2) of the FLIM data. This acquisition time is approaching that compatible with in vivo and time-lapse imaging and we are now working towards demonstrating FLIM FRET readouts in live subjects. In the future, we hope to reduce the effects of sample motion during in vivo FLIM acquisition through the use of “FIFO” FLIM combined with software-based detection and correction of image motion during the acquisition. We note that there is a trade-off between image speed and spatial resolution that should be optimised for specific applications.

## 3. Conclusions

Our intention has been to demonstrate the broad applicability of FLIM to read out FRET in a range of applications across the spectrum of the drug-discovery pipeline. FLIM FRET is well established in microscopy where it has almost become a standard tool to study biomolecular interactions. To date, however, there has been limited translation to high-throughput assays or to in vivo imaging. We have demonstrated that it is possible to construct an automated FLIM multiwell-plate reader that can acquire optically sectioned lifetime images of fixed and live cells labelled with fluorescent proteins in a few seconds per field of view and read an entire 96-well plate in less than ≈15 min. Although our time-gated imaging instrumentation utilises relatively long time gates of 2–3 ns duration to maximise the number of detected photons, it is still capable of determining fluorescence lifetimes of less than ∼100 ps and of resolving lifetime differences of ∼20 ps. Using global analysis, the system can accurately determine the ratio of contributions from different lifetime components, which may be used to quantify mixtures of fluorophores or to calculate the fraction of donor molecules undergoing FRET.

As an exemplar application, we have applied this instrument to study the aggregation of HIV-1 Gag proteins in VLP formation at the cell membrane, which is challenging because the unknown stoichiometry of the fluorescent protein expression can mean that the average change in the donor fluorescence lifetime over the field of view is rather low, that is, a few hundred picoseconds. The FLIM readout of this FRET assay can be improved by the use of image segmentation and global analysis such that the expected change in CFP lifetime for an assay of compounds inhibiting Gag protein aggregation at the membrane would be closer to 350 ps.

The information available from FLIM FRET studies of cell-signalling networks could be further enhanced by multiplexing readouts, for example, of multiple protein interactions. While most FLIM and FRET experiments are applied to study a single component of a cell-signalling network, we have shown that multiplexed FLIM can be applied to read out changes in the lifetime of two fluorophores in a single time-lapse acquisition. We aim to implement this facility in our automated FLIM multiwell-plate reader where it could provide increased specificity for screens and systems biology.

While there are many alternative approaches to read out FRET of protein interactions, the robust nature of fluorescence lifetime measurements makes FLIM an attractive approach to translate cell-based assays to live organisms. We have shown that FLIM can be implemented in a confocal endomicroscope, thus permitting signalling processes in cells to be studied in vivo in intact animals. This could permit longitudinal studies in mammals, greatly reducing the numbers of animals required for testing, for example for drug discovery, and improving the value of the multiple readouts compared to measurements on multiple animals sacrificed at different endpoints. Further reduction in the numbers of such animal tests may be achieved if more physiological data can be obtained from assays in simpler organisms such as zebrafish. There is increasing interest in such assays, and technology for automated high-throughput imaging of zebrafish is being developed.[72] We have demonstrated the first FLIM of fluorescent proteins in zebrafish and shown that (FLIM) OPT can be applied without chemical clearing. This opens the path to FLIM-OPT of live zebrafish for FRET readouts of spatiotemporal dynamics of signalling networks in vivo.

## Experimental Section

**Figure 10:**
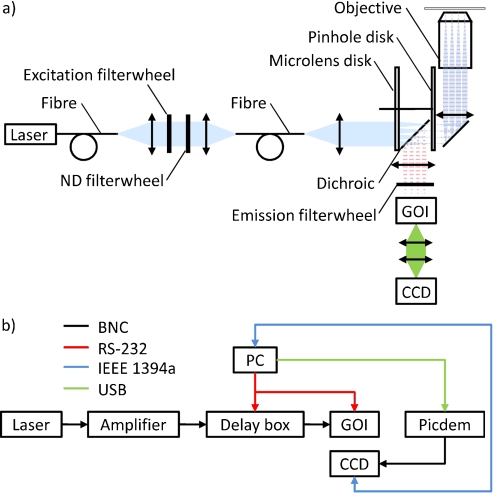
Schematics of a) optical and b) electronic configurations of an optically sectioned FLIM multiwell plate reader based on a modified IN Cell 1000 Analyser.

The FLIM and FRET data presented here was obtained using wide-field time-gated imaging and laser scanning TCSPC.[21] The time-gated imaging was implemented using gated optical image intensifier (GOI) technology, for which we have recently characterised the signal-to-noise performance.[73] In order to accurately fit the fluorescence decay data, temporal IRFs were recorded using solutions of DASPI or erythrosin B, which have fluorescence lifetimes shorter than 150 ps. These FLIM techniques were implemented in the following instruments:

*Automated Nipkow FLIM Multiwell Plate Reader:* Figure 10 presents the optical and electronic configurations of the FLIM multiwell plate reader, which is based on a commercial plate reader (IN Cell Analyzer 1000, GE Healthcare) that is built around a wide-field microscope and incorporates an optical autofocus module and motorised sample stage.

To implement optically sectioned FLIM, we utilised the high-speed Nipkow FLIM instrumentation described in Grant et al.[27] but incorporated a newly available Nipkow disc head (Yokogawa, model CSU-X M1L) that provides improved light efficiency, particularly of the excitation radiation, compared to the unit (Yokogawa CSU-10) used previously.[27,28] Because the Nipkow unit is self-contained with respect to the excitation and fluorescence light paths, it is straightforward to implement via a camera port of a wide-field microscope. This FLIM multiwell plate reader was controlled by in-house software written in LabVIEW (National Instruments) that automatically moves the well plate, focuses the microscope, finds cells and can set the FLIM acquisition parameters in a manner similar to our previous automated plate reader.[28] Excitation was provided by a frequency-doubled femtosecond Ti:Sapphire laser (Spectra-Physics, model Tsunami) or by a fibre-laser-pumped supercontinuum source (Fianium UK Ltd, SC400-6) that produces ∼10 ps pulses at 60 MHz repetition and ∼2 mW nm^−1^ average spectral power density with sufficient spectral coverage to excite CFP, YFP and mCherry etc. Electronically controlled wavelength selection was implemented using a motorised filter wheel. Time-gated imaging was provided by a gated optical intensifier (Kentech Instruments, model HRI) that was customised to provide time-gates as long as 3 ns. This was read out using a cooled CCD camera (Hamamatsu Photonics, model ORCA-ER). The HRI was operated with 2–3 ns-wide gates triggered at various delay times after the excitation pulses to temporally sample the fluorescence decay profiles for each pixel in the field of view. Typically ∼7 gate delays, with ∼1 s CCD integration time at each delay, are used to sample fluorescence from labelled cells. A PicDem circuit (Microchip Technology, Inc.) was used to provide a trigger for the CCD camera and can also be used to control a shutter that blocks the excitation beam between acquisitions in order to minimise photobleaching.

For automated image acquisition, the user inserts the multiwell plate sample array, selects which wells to image and sets the delay of the initial time gate with respect to the excitation pulses and an intensity threshold for the cell seeking routine. In order to minimise the time required to read a multiwell plate we used a “prescan” mode, which uses a seek algorithm to find specific number of fields of view that have a predetermined fraction of pixels above a given threshold. This information is used when we then run a “full scan” to acquire the desired FLIM data. It is also possible to undertake a rapid FLIM prescan, acquiring only two or three time-gates per image, in order to automatically determine the delays of the time-gates sampling the fluorescence decay profiles for the full scan.

Following the automated data acquisition, fluorescence data were fitted to decay models that were convolved with the temporal instrument response function (IRF) using in-house software written in LabVIEW (National Instruments) designed to provide fluorescence lifetime maps and fluorescence intensity images (calculated by integrating under the decay curve). We typically represent fluorescence lifetime data in fluorescence lifetime “images” for which a false colour scale encodes the lifetime parameter and the brightness encodes the total fluorescence intensity. We note that incorporating an appropriate IRF in the fitting procedure is essential for accurate fitting of complex decay profiles. Our global fitting programme can also take account of incomplete fluorescence decays at the arrival of the “next” excitation pulse. This software is able to automatically batch-process the data from multiple wells.

For rapid multiwell plate imaging, we typically record each image with seven time gates and, for an average excitation power of less than 1 mW at the sample, acquisition times are typically <10 s per image, permitting us to read a 96-well plate in less than 20 minutes and deliver fluorescence lifetime images fitted pixelwise to monoexponential decay profiles. These image acquisition times are often too short to record sufficient photons to accurately fit complex exponential decays on a pixel by pixel basis. When we wish to analyse our data with respect to complex decay models, we can either increase the image acquisition times to 10–100 s per field of view in order to detect sufficient photons per pixel or we can acquire fluorescence data as for monoexponential decay fits and apply image segmentation and binning to reach a sufficient number of detected photons. Alternatively, we can apply “global fitting”, with or without image segmentation, to derive parameters from complex decay models with a similar number of detected photons per pixel as is required for fitting to monoexponential decay models.

Our in-house image segmentation and global fitting software is written in C++ with a MATLAB front end and will be described in detail in a future publication. We note that traditional nonlinear fitting approaches are not appropriate for global fitting of FLIM data as the number of non-linear parameters is of the order of the number of pixels, which is generally rather large. To make the global analysis problem computationally tractable we use a Variable Projection algorithm as described by Kaufman[74] and discussed further by Golub and Pereyra[75] combined with a standard Levenberg–Marquardt nonlinear least squares (NLLS) solver. We first note that the expression for a multiexponential decay as described by Equation (1) may be expressed as a linear combination of nonlinear functions (i.e., the exponential functions). For the global analysis problem the nonlinear functions depend only on the lifetimes and so are constant across the image. For a given set of nonlinear parameters, the linear parameters (the pre-exponential factors) may be determined by solving a set of linear equations; since the pre-exponential factors for a given pixel only depend on the data recorded in that pixel, the linear least squares problem may be partitioned into a set of independent small least-squares problem for each pixel. Exploiting this knowledge, we may reduce the dimensionality of the NLLS problem to the number of exponentials. We use a modified version of the Variable Projection algorithm implemented by LeVeque (http://www.netlib.org/opt/varpro) adjusted such that the memory requirements scale linearly rather than quadratically with the number of pixels. It should be noted that the main computational burden of traditional pixel-by-pixel NLLS fitting of FLIM data lies in the calculation of the model function at each iteration. Therefore, since the global implementation as described only evaluates the model function once per iteration for the entire image, it is significantly less computationally intensive that the pixel-by-pixel approach. The memory requires are higher as all pixels must be kept in memory simultaneously. As an example, a global fit across six images in the GFP-mCherry data shown in Table 1 took 0.5 s on a workstation with a 2.9 GHz Intel Core i7 processor while using 400 Mb of memory for data processing.

**Figure 11:**
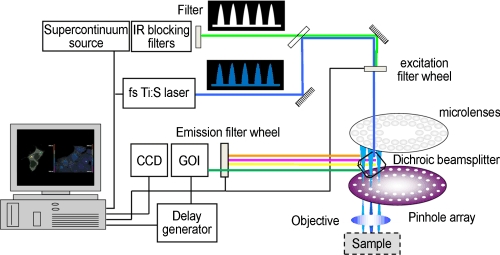
Experimental setup for multiplexed optically sectioned FLIM microscopy.

*Multiplexed Optically Sectioned FLIM Microscope:* This microscope utilised essentially the same experimental setup as described in ref. [27] except that the manually configurable Nipkow disc unit (Yokogawa CSU-10) was replaced by the newly available Nipkow disc unit (Yokogowa, model CSU-X A3) that provides improved light efficiency and electronic switching of the dichroic beam splitters (Figure 11). By introducing electronically controlled filter wheels for the excitation and emission pathways and synchronising these to the dichroic changer and the FLIM acquisition hardware, we were able to automatically switch excitation and detection wavelengths in order to image different fluorophores. For excitation we used a fibre laser-pumped supercontinuum source (Fianium Ltd, model SC450-6) and a frequency-doubled femtosecond Ti:Sapphire laser (Spectra-Physics, model Tsunami). The computer-controlled hardware was able to switch between configurations optimised for different fluorophores in about one second, enabling us to acquire interleaved series of time-gated FLIM acquisitions. Thus, we were able to multiplex two FLIM readouts, sequentially acquiring interleaved time-lapse FLIM data. This can be applied to read out, for example, two FLIM FRET measurements of FRET biosensors or intermolecular FRET reporting protein–protein interactions.

**Figure 12:**
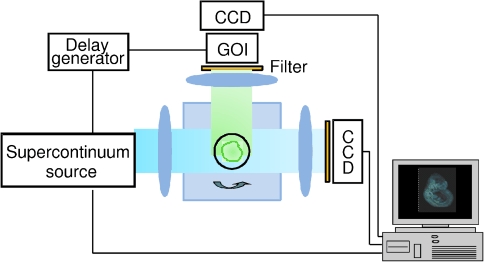
Schematic of the experimental configuration for FLIM OPT studies.

*FLIM OPT Instrumentation:* Figure 12 shows a schematic of the experimental setup for wide-field time-gated tomoFLIM. The sample is suspended beneath an electronically controlled rotation stage in a reservoir of index matching fluid. A fibre laser-pumped super-continuum source (Fianium SC 450-6) was used to excite the fluorescence, which was imaged onto the GOI of a wide-field time-gated imaging system and the resulting time-gated and intensified fluorescence signals were recorded by a CCD camera, in a similar manner described earlier for FLIM.

It is also possible to record the transmitted excitation radiation in order to reconstruct a 3D map of the absorption at the excitation wavelength. The sample is rotated about the *Z* axis and typically a series of time-gated images are recorded at regular intervals over a full 360° rotation for each setting of the time gate delay. 3D maps of fluorescence intensity and lifetime are then reconstructed using filtered back-projection.[76] At each value of the time gate delay, a 1D Fourier transform is applied to each projection in a particular *X*–*Y* plane of the sample and the resulting functions are then filtered in the Fourier domain to account for unequal sampling of the spatial frequency components. The inverse Fourier transform is then applied and the resultant filtered projections are backprojected at the corresponding angle to reconstruct the *X*–*Y* (time-gated) intensity distribution of the sample in that plane. The fluorescence lifetimes are then calculated in the usual way by fitting the series of time-gated fluorescence intensity images to an appropriate decay model at each *X*–*Y* plane to obtain the fluorescence lifetime map or image for that plane. To visualise the whole sample, we typically use 3D rendering software (Volocity Visualisation, PerkinElmer) applied to the *Z*-stack of fluorescence lifetime images.

**Figure 13:**
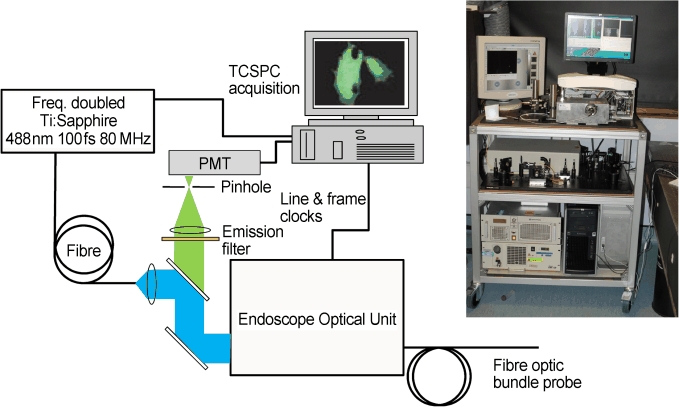
Schematic of a confocal FLIM endomicroscope with inset photograph of the instrument mounted on a clinically deployable trolley.

*Confocal FLIM Endomicroscope:* The confocal FLIM endomicroscope (Figure 13) was based on a commercially available endomicroscope (Cellvizio® GI, Mauna Kea Technologies), which is a laser scanning single-photon fluorescence fibre bundle endomicroscope that is capable of recording optically sectioned images with a variety of fibre probes. For the experiments reported here, we used a coherent bundle containing 30 000 optical fibre cores that was terminated with a miniature objective (Cellvizio® Mini O). The probe has a lateral resolution of ∼1.4 µm, a working distance of 60 µm and a field of view of 240 µm. The diameter of the distal tip is 2.6 mm.

For FLIM of live cells labelled with EGFP, excitation pulses centred at 481 nm from a fibre-delivered mode-locked frequency-doubled Ti:Sapphire laser (Spectra Physics, model Mai-Tai) were coupled into the internal beam path of the endoscope immediately before the scanning assembly and the descanned fluorescence was focussed through a pinhole onto a photomultiplier that was connected to the TCSPC card (SPC830, Becker & Hickl GmbH) for FLIM.[53] A 505 nm dichroic filter was used in combination with a 520–550 nm bandpass emission filter to select the fluorescence from the EGFP. The internal resonant scanner was operated in bidirectional mode, resulting in two mirrored images—one for each scan direction. A single interlaced image was produced with an automated procedure using the cross correlation of the two images to provide a final output image size of up to 350 x 512 pixels, which is limited by the TCSPC card memory. For faster imaging, the FLIM data could be binned to increase the effective number of photons for fitting. The line and frame synchronization signals were obtained from the endoscope electronics and the pixel clock (300 ns period) was derived from the TSCPC card. FLIM images were calculated using SPCImage (Becker and Hickl, GmbH) from TCSPC data obtained for exposure durations varying from 0.2 to 60 s with an average power of 360 µW over the field of view, which resulted in a typical pixel count rate of ∼3–7 x 10^5^ counts s^−1^.

**Sample Preparation:** *Dye Samples:* The dye mixtures of Rhodamine B and Rhodamine 6G were prepared by dissolving pure dye powder into spectral grade methanol and then subsequent dilution in MilliQ purified water (Millipore, Billerica, USA) to give a final 10 μm solution of each dye. The Rhodamine B and Rhodamine 6G solutions were mixed in ratios of 100:0, 80:20, 60:40, 40:60, 20:80 and 0:100 and imaged using the automated Nipkow FLIM multiwell plate reader, which used a 40x CFI PLAN Fluor ELWD 0.60 NA objective (Nikon, Tokyo, Japan) and a Di01-T488/532 dichroic beamsplitter (Semrock, Rochester, USA). The dataset was acquired using six time-gated images with a width of 2000 ps. Analysis of lifetime data was conducted using an in-house lifetime fitting software as described above.

The DASPI samples in a composite solvent were prepared by the vigorous mixing of glycerol and ethanol, followed by the addition of DASPI (prepared by dissolving DASPI powder into spectral grade methanol) and further vigorous mixing. These samples were imaged with a 465/30 nm excitation filter, a Di01-T488 dichroic beamsplitter and a 525/50 nm emission filter (Semrock, Rochester, USA).

*Cell Culture and Transfection Systems:* HEK293T and COS-7 cells were grown as a monolayer at 37°C, 5% CO_2_ in DMEM (Gibco, Invitrogen) with high glucose and supplemented with 10% fetal bovine serum and 0.5% penicillin/streptomycin.

For Gag expression, HEK293T cells were seeded the day prior to transfection, in 25 cm^2^ flasks and grown overnight until >70% confluent. The following day, transfections were carried out using Effectene transfection reagent (Qiagen, Germany) following the manufacturer’s protocol. Cells were incubated with the transfection mixture for eight hours at 37°C, 5% CO_2_, then washed with phosphate buffer, trypsinized, plated into 96 well plates, and incubated in fresh media at 37°C, 5% CO_2_ until next day, when they were imaged.

For EGFP and EGFP-mCherry constructs, transient transfection was used for the HEK293T cells. 20 µl of the transfection mixture was added to each well of the plate and 175 µl of cells were laid on top of the transfection mixture. The plate was then incubated overnight at 37°C and 5% CO_2_. Before imaging, the media was removed, the cells were washed with phosphate buffer and Hank’s balanced salt solution was added. COS-7 cells used for microconfocal FLIM endoscopy were plated on Matek (USA) glass bottom Petri dishes and transfections were carried out using Effectene. Images were taken the following day.
